# Expression of ABCG2 and Bmi-1 in oral potentially malignant lesions and oral squamous cell carcinoma

**DOI:** 10.1002/cam4.182

**Published:** 2014-01-11

**Authors:** Andrew J Dalley, Luke P Pitty, Aidan G Major, Ahmad A AbdulMajeed, Camile S Farah

**Affiliations:** 1 UQ Centre for Clinical Research, The University of QueenslandHerston, Qld, 4029, Australia; 2 School of Dentistry, The University of QueenslandBrisbane, Qld, 4000, Australia

**Keywords:** ABCG2, biomarkers, Bmi-1, cell lines, head and neck cancer, oral epithelial dysplasia

## Abstract

Early diagnosis is vital for effective treatment of oral squamous cell carcinoma (OSCC). The optimal time for clinical intervention is prior to malignancy when patients present with oral potentially malignant lesions such as leukoplakia or erythroplakia. Transformation rates for oral dysplasia vary greatly and more rigorous methods are needed to predict the malignant potential of oral lesions. We hypothesized that the expression of two putative stem cell markers, ABCG2 and Bmi-1, would correlate with disease severity for non diseased, potentially malignant and OSCC specimens and cell lines derived from an equivalent range of tissues. We compared immunoreactive protein and relative gene expression of ABCG2 and Bmi-1 in eight cell lines derived from source tissues ranging in disease severity from normal (OKF6-TERT2) through mild and moderate/severe dysplasia (DOK, POE-9n) to OSCC (PE/CA-PJ15, SCC04, SCC25, SCC09, SCC15). We also analyzed immunoreactive protein expression of ABCG2 and Bmi-1 in 189 tissue samples with the same range of disease severity. A trend between oral lesion severity to ABCG2 and Bmi-1 immunostain intensity was observed. Flow cytometry of oral cell lines confirmed this trend and gave good correlation with RT-PCR results for ABCG2 (*r *= 0.919, *P *= 0.001; Pearson) but not Bmi-1 (*r *= −0.311). The results provide evidence of increased density of ABCG2 and Bmi-1-positive populations in malignant and oral potentially malignant lesions and derived cell lines, but that intragroup variability within IHC, flow cytometry, and RT-PCR results compromise the diagnostic potential of these techniques for discriminating oral dysplasia from normal tissue or OSCC.

## Introduction

Early diagnosis is of paramount importance for effective treatment of oral squamous cell carcinoma (OSCC), the incidence of which is rising in the developed world among a younger demographic that have less association with known risk factors such as excessive alcohol or tobacco consumption 1. The optimal time for clinical intervention is prior to malignancy when patients present with oral potentially malignant lesions (OPML) such as oral leukoplakia or erythroplakia. The presence and severity of dysplasia in OPMLs is associated with poorer clinical outcomes 2,3, but more rigorous methods are needed to predict the malignant potential of oral lesions as rates of malignant progression from oral dysplasia vary greatly 4.

Tumors often contain hierarchical arrangements of genetically or epigenetically dissimilar tumorigenic and non tumorigenic cells 5–7. In these cases, a theoretic subpopulation of tumorigenic cells termed Cancer Stem Cells have been identified through detailed investigations with disaggregated tumor-derived cells, NOD/SCID mouse transplant models 8 and antigenic 7,9,10 markers of cell phenotype. The acquisition of a genetic or epigenetic predisposition to tumorigenicity by cancer stem cells is a cumulative process 5 that may be evident in premalignant lesions. The association of putative stem cell antigens with oral potentially malignant lesions 11,12 and oral epithelial tumorigenicity 10 is the subject of a recent review 13.

A recent long-term follow-up study of 135 patients associated two stem cell markers, ATP-binding cassette G2 subfamily (ABCG2) and Bmi-1 with transformation of oral leukoplakia to cancer 2. ABCG2 belongs to the ATP-binding cassette transporter superfamily that contributes to multiple drug resistance in cancers and permits cells in vitro to export the fluorescent dye Hoechst 33342, a technique used to define a side population of purported stem cells 13–16. Bmi-1 (B lymphoma Mo-MLV insertion region 1 homolog) contributes to the epigenetic regulation of cell cycle and senescence of tissue stem cells via the INK4A loci 17. Bmi-1 is oncogenic and has been associated with purported cancer stem cells and cell lines derived from head and neck squamous cell carcinoma 10,13,16,18–22.

We hypothesized that ABCG2 and/or Bmi-1 expression would correlate with disease severity for a collection of cell lines derived from non diseased, potentially malignant, and OSCC specimens, and that these correlations would also hold for patient samples with a similar range of oral disease severity. Our aim was to ascertain whether in vitro analysis with established cell lines provides a valid model for further investigation of the mechanistic involvement of ABCG2 and/or Bmi-1 in neoplastic transformation of oral dysplasia to OSCC, and to evaluate whether immunohistochemical analysis of ABCG2 and/or Bmi-1 has diagnostic potential for discriminating oral dysplasia from normal tissue or OSCC. Accordingly, we compared ABCG2 and Bmi-1 antigen positive population densities among eight cell lines by flow cytometry, correlated this with RNA expression analysis of the gene products, and conducted a confirmatory retrospective cohort study of ABCG2 and Bmi-1 antigen stain intensity in 189 patient samples.

## Material and Methods

### Cell lines

Cell lines were purchased from the European Collection of Cell Cultures (ECACC) (Sigma, St Louis, MO), the Rheinwald Laboratory (Harvard Medical School) and the American Type Culture Collection (ATCC). Cells were cultured according to the supplier's instructions unless otherwise specified 23. Human neoplastic oral keratinocytes (PE/CA-PJ15, ECACC accession no. 961211230, received at passage 7), were grown in Iscove's modified Dulbecco's medium (IMDM) (#12440; Invitrogen, Grand Island, NY) containing 4 mmol/L L-glutamine, 10% fetal calf serum (FCS) (#10099; Invitrogen), 100 U/mL of penicillin, 100 *μ*g/mL of streptomycin, and 250 ng/mL of amphotericin B (Antibiotic-Antimycotic) (#15240; Invitrogen). Human dysplastic oral keratinocytes (DOK, ECACC accession no. 94122104, received at passage 23), were grown in Dulbecco's modified Eagle's medium (DMEM) (#11995; Invitrogen) supplemented with 10% FCS, 10.3 *μ*mol/L hydrocortisone (#H2270; Sigma) and Antibiotic-Antimycotic. Human severe dysplastic oral keratinocytes (POE-9n, received at passage 4) and hTERT transfection immortalized normal human keratinocytes (OKF6-TERT2, received at passage 16) were grown in keratinocyte serum-free medium (Invitrogen K-sfm) (#17005-042; Invitrogen) containing 25 *μ*g/mL bovine pituitary extract (BPE) (#13028-014; Invitrogen) and 0.2 ng/mL human recombinant epidermal growth factor 1-53 (EGF 1-53) (#10450-013; Invitrogen), supplemented with CaCl_2_ solution to a final concentration of 0.4 mmol/L and with Antibiotic-Antimycotic. Human neoplastic oral keratinocytes (SCC04 cell line, ATCC lot no. 58078512, received at passage 13) were grown in DMEM supplemented with 400 ng/mL hydrocortisone and 10% FCS. Human neoplastic oral keratinocytes (SCC09 cell line, ATCC lot no. 58078572, received at passage 14; SCC15, ATCC lot no. 58483182, received at passage 19; SCC25, ATCC lot no. 58078571, received at passage 14) were grown in 1:1 mixture of DMEM and Ham's F12 medium with 2.5 mL L-glutamine adjusted to contain 15 mmol/L HEPS, 0.5 mL sodium pyruvate, 1.2 g/L sodium bicarbonate, and supplemented with 10% FBS and 400 ng/mL hydrocortisone. Cells were photographed with phase contrast at ×10 magnification while growing inside a humidified CO_2_ gassed incubator at 37°C using an Incucyte-FLR™ cell imaging platform (Essen BioScience, Ann Arbor, MI).

### Multicolor flow cytometry

Flow cytometry was performed on discrete replicate cultures (*n *=* *3) of OKF6-TERT2, DOK, POE-9n, PE/CA PJ15, SCC04, SCC25, SCC09 and SCC15. Subconfluent OKF6-TERT2 (passage 20, 25, 26), DOK (passage 40-42), POE-9n (passage 7, 7, 16), PE/CA PJ15 (passage 21, 26, 27) SCC04 (passage 21-23), SCC25 (passage 18-20), SCC09 (passage 20-22), and SCC15 (passage 25, 25, 26) cells were detached from 75 cm^2^ tissue culture flasks (#430641; Corning Inc, Lowell, MA) with 8 ml TrypLE™ Express (#12604; Invitrogen), collected into media, pelleted (by centrifugation at 400 g for 5 min), and resuspended in phosphate-buffered saline (PBS; #E404; Amresco, Solon, OH). 1 × 10^6^ cells were washed twice with PBS and stained with LIVE/DEAD® Fixable Aqua Dead Cell Stain Kit (#L34957; Invitrogen), fixed with 4% neutral buffered formalin (#HT501128; Sigma), washed with PBS and permeabilized with permeabilization buffer consisting 0.1% w/v saponin (#S-4521; Sigma), 4% fetal calf serum (#10099; Invitrogen) in PBS. Fixed/permeabilized cells in 100 *μ*L of permeabilization buffer were exposed for 1 h at 4°C in the dark to combinations of 5 *μ*L of APC-anti-human ABCG2 (#561451; clone 5D3, BD Bioscience, San Jose, CA), 5 *μ*L Anti-human Bmi-1 Fluorescin Monoclonal Antibody (#IC33341F; clone 384515, R&D Systems, Inc, Minneapolis, MN) or isotypes controls: 5 *μ*L APC Mouse IgG2b *κ* (#555745; clone 27-35, BD Bioscience) and 5 *μ*L Mouse IgG_2A_ Isotype control-CFS (#IC003F; 20102, R&D Systems Inc). Labeled cells were washed with permeation buffer, then PBS, and finally resuspended in 400 *μ*L of Flow buffer consisting: 2 mmol/L EDTA (#E5134; Sigma), 0.5% BSA (#A9418; Sigma), and 1% w/v sodium azide (#S2002; Sigma) in PBS. Sample analysis was performed on a Gallios 10-color 3-laser flow cytometer (#775106; Beckman Coulter, Brea, CA). One hundred thousand events were recorded for each sample. Data analysis was performed using Kaluza™ software (version 1.2; Beckman Coulter).

### Semi-quantitative real-time polymerase chain reaction (RT-PCR)

RNA extraction used TRIzol® reagent (#15596018; Invitrogen), phase lock tubes (#2302810, 5Prime Inc., Gaithersburg, MD), chloroform (#C-2432, Sigma), and the PureLink RNA mini-kit (#12182; Invitrogen) with on-column DNA digestion using PureLink™ DNase (#12185010; Invitrogen). Synthesis of cDNA used the SuperScript® III First-Strand Synthesis SuperMix kit (# 18080-400; Invitrogen). Primers (GeneWorks Pty Ltd., Thebarton, SA, Australia) were designed to cross intron/exon boundaries: RPL13A, forward 5′-ATGGTCGAGGCCATCTCCTG-3′, reverse 5′-TGATGCCTTCACAGCGTACG-3′; ABCG2, forward 5′-CGCATCCTGAGATCCTGAGCC-3′, reverse 5′ TCGACATTACTGGAAGACATCTGG 3′; Bmi-1, forward 5′-TGTCTTTTCCGCCCGCTTCG-3′, reverse 5′-TCTCGT TGTTCGATGCATTTCTGC-3′. RT-PCR and product melt curves were performed with a StepOnePlus Real-Time PCR System (Applied Biosystems®, Invitrogen). Optimized reactions (15 *μ*L) contained 7.5 *μ*L SYBR® Green PCR Master Mix (#4309155; Invitrogen), 0.15 *μ*L primers at 20 pmol/*μ*L, 1.5 *μ*L diluted cDNA, and 5.7 *μ*L UltraPure™ water (#10977015; Invitrogen). Thermocycling: 50°C for 2 min then 95°C for 2 min followed by 60 cycles of: 95°C for 15 sec, 60°C for 30 sec, with observed melts at 95°C for 15 sec then 60°C for 1 min. Reported *C*_*t*_ values were determined from duplicate assays within two discrete RT-PCR runs (giving *n *= 4 *C*_*t*_ values). The comparative ΔΔ*C*_t_ method was used to quantify target mRNA relative to the endogenous reference RPL13A and the cell line reference OKF6-TERT2.

### Patient samples

Of 189 formalin-fixed paraffin-embedded (FFPE) specimens (80 male, 61 female and 48 gender not recorded; mean age 54 years, range 18–86 years) comprised: 74 normal oral mucosa (referred to herein as normal), 17 mild dysplasia (MD), 17 moderate to severe dysplasia (SD), and 81 oral squamous cell carcinoma (OSCC). Sample diagnosis was confirmed retrospectively by an oral pathologist (CSF) according to the World Health Organization (WHO) classification system 24. The study received ethical review and approval from Hospital and University Human Research Ethics Committees (HREC/10/QRBW/336 and UQ/2007001478).

### Immunohistochemistry

Immunohistochemistry (IHC) was performed as described previously 12,23,25. Briefly, 5-*μ*m sections were incubated overnight at 4°C with primary antibody diluted in Background Sniper (#BS966M, Biocare Medical, Concorde, CA, USA). Primary antibodies: ABCG2 (BXP-21) (#SC-58222, Santa Cruz Biotechnology Inc., Dallas, TX) at 1:500 dilution, Bmi-1 (1.T.21) (#ab14389, Abcam plc., Cambridge, UK) at 1:100 dilution. Staining utilized MACH 1 Mouse Probe, MACH 1 Universal HRP-Polymer (#M1U539L10; Biocare Medical) with Betazoid DAB chromogen (#BDB2004L; Biocare Medical), CAT haematoxylin (#CATHE-M; Biocare Medical), and Leica CV Mount (Leica, Nussloch, Germany). Positive and negative staining controls for each antibody were conducted (Figure S1).

### IHC Scoring

Ten random fields were scored at ×400 magnification, counting >500 cells. Sections were scored for stain intensity as described previously 12,25 (scaled as: 0 = no stain to 4 = dark brown) and the percentage of positively stained epithelial cells (categorized as: 0% = score 0; <25% = score 1; 25–49% = score 2; 50–74% = score 3; and 75–100% = score 4) was calculated. The final index score, which ranged from 0 to 16, was the product of categorized percentage positive score and the scaled stain intensity score.

### Statistical analysis

Nonparametric statistical analysis was performed using IBM SPSS Statistics V.20 software (IBM Corporation, Armonk, NY) and the R computing language was used to compile plots (R Development Core Team [http://www.R-project.org]). Median (Q2) and inter-quartile range (IQR) or mean (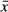) and standard deviation (SD) have been presented. Immunolocalization score data for disease groups were compared to normal using Mann–Whitney *U* tests with Bonferroni correction for *n *= 3 comparisons (*P *< 0.016). Pearson product-moment correlation coefficients were used to compare average real-time PCR results with average flow cytometry results.

## Results

### ABCG2 and Bmi-1 antigen on normal, dysplastic, and OSCC-derived cell lines

**Figure 1:**
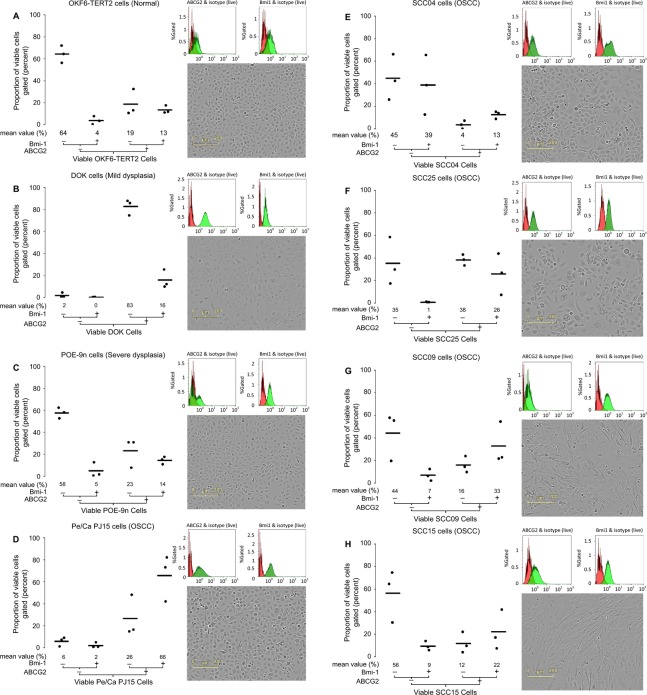
Expression of ABCG2 and Bmi-1 in normal, dysplasia and SCC-derived oral cell lines by flow cytometry, and comparison of cell morphology. Strip plots showing co-expression data for ABCG2 and Bmi-1 positive (+) and negative (−) cell populations after gating for live cells within (A) OKF6-TERT2, (B) DOK, (C) POE-9n, (D) PE/CA PJ15, (E) SCC04, (F) SCC25, (G) SCC09, and (H) SCC15 submerged monolayer cellcultures. The strip plots show mean (line) and values of n = 3 independent experiments over a range of cell passages. Plots are identified by a co-expression tree and mean values ≥1% are given below each plot. Adjacent to each box and whisker plot are corresponding fluorescence intensity histograms for viable cells stained with direct conjugated antibodies against ABCG2 (upper left panel) and Bmi-1 (upper right panel; shown in green) overlaid with the corresponding histograms of cells stained with appropriate isotype control antibodies (shown in red); data from one representative experiment shown. Also adjacent to each box and whisker plot (lower right panel) are phase contrast photographs of each cell line taken via an Incucyte-FLRTM within incubator cell imaging platform at 910 magnification.

Expression of ABCG2 and Bmi-1 was rigorously evaluated in a collection of eight oral cell lines originating from normal tissue (OKF6-TERT2), mild dysplasia (DOK), severe dysplasia (POE-9n), and oral squamous cell carcinoma (PE/CA PJ15, SCC04, SCC25, SCC09, and SCC15). Mean percent expression data for ABCG2 and/or Bmi-1 antigen expression by viable cells are presented in Figure 1, and are derived from three discrete replicate experiments over a range of cell passages. Supportive histograms that overlay positive and isotype control fluorescence intensities are provided in Figure 1, along with comparative in situ photographs of each cell line under post log phase growth conditions.

With the exception of SCC09 and SCC15, all of the cell lines exhibit characteristic epithelial morphology and growth features, forming monolayers with squamous appearance, restricted intercellular space, regimented intercellular cohesion, and clonal growth. SCC09 and to a lesser extent SCC15 exhibited more of a mesenchymal morphology, being multipolar with greater intercellular space and exhibited a dispersed growth pattern (Fig. 1).

Normal oral tissue-derived cell populations (OKF6-TERT2) were predominantly negative for both ABCG2 and Bmi-1 (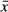  = 64% ± 6%), and contained higher proportions of ABCG2 positive cells (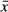  = 32% ± 8%) than Bmi-1 positive cells (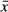  = 17% ± 6%), (Table 1). Predominance of ABCG2 positivity over Bmi-1 positivity was also observed for both dysplasia-derived cells (DOK and POE-9n) and two of the five OSCC-derived cells (PE/CA PJ15 and SCC25), (Table 1). Only one cell line (SCC04) contained higher proportions of Bmi-1 positive cells than ABCG2 positive cells, while the two remaining cell lines (SCC09 and SCC15), which exhibit the least epithelial-like phenotype, exhibited approximately equal proportions of ABCG2 and Bmi-1-positive cells (Table 1). The mild dysplasia-derived cell line (DOK) was exceptional in exhibiting approximately 100% ABCG2 positivity (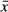 = 98% ± 2%), (Table 1, Fig. 1 & supporting histogram), closely followed by an OSCC-derived cell line (PE/CA PJ15), which contained mean 92% ± 4% ABCG2-positive cells (Table 1). All five OSCC-derived cell lines exhibited greater proportions of Bmi-1-positive cells than the normal tissue and dysplasia-derived cells (Table 1). One OSCC-derived cell line (PE/CA PJ15) was exceptional in containing far higher proportions of dual positive (ABCG2 and Bmi-1-positive) cells (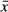 = 66% ± 17%) than any of the other cell lines (Table 1). With the exception of SCC04, all of the OSCC-derived cells contained higher proportions of dual positive cells than the normal tissue and dysplasia-derived cells (Table 1).

**Table 1:** Mean (SD) percentage of viable cell population gated upon ABCG2 and Bmi-1 expression in normal, dysplasia and SCC-derived oral cell lines by flow cytometry.

	Mean (SD) percentage of viable cell population gated			
	ABCG2 −ve/Bmi1 −ve	ABCG2 + ve	Bmi1 + ve	ABCG2 + ve/Bmi1 + ve
OKF6-TERT2	64 (6)	32 (8)	17 (6)	13 (3)
DOK	2 (2)	98 (2)	16 (7)	16 (7)
POE-9n	57 (4)	37 (9)	19 (8)	14 (3)
PE/CA PJ15	6 (3)	92 (4)	68 (18)	66 (17)
SCC04	45 (16)	16 (5)	51 (19)	13 (3)
SCC25	35 (17)	64 (18)	27 (15)	26 (15)
SCC09	44 (17)	49 (21)	40 (12)	33 (15)
SCC15	56 (19)	34 (22)	32 (11)	22 (14)

### ABCG2 and Bmi-1 gene expression in normal, dysplastic, and OSCC-derived cell lines

**Figure 2:**
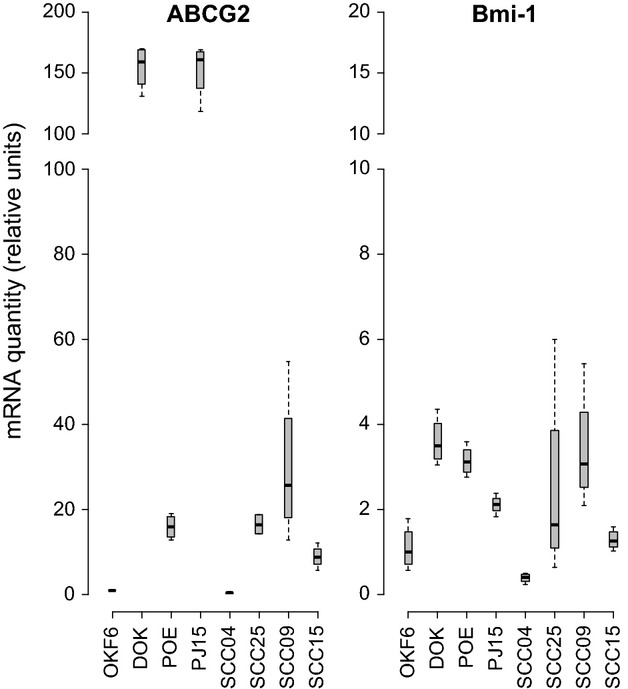
Box and Whisker plots of relative mRNA expression values for ABCG2 and Bmi-1 in oral mucosa cell lines derived orriginally from normal (OKF6-TERT2), mild dysplasia (DOK), severe dysplasia (POE-9n), and OSCC (PJ15, SCC04, SCC25, SCC09, SCC15) tissues. Cycle threshold (*C*_*t*_) values for mRNA expression are presented relative to the reference cell line, OKF6-TERT2. Abreviated names for OKF6-TERT2, POE-9n and Pe/Ca PJ15 have been used.

Semi-quantitative real-time PCR analysis of ABCG2 and Bmi-1 gene expression in eight oral cell lines originating from normal tissue (OKF6-TERT2), mild dysplasia (DOK), severe dysplasia (POE-9n), and oral squamous cell carcinoma (PE/CA PJ15, SCC04, SCC25, SCC09 and SCC15) are provided in Figure 2 and Table 2. Good correlation between relative quantity of mRNA for ABCG2 (Table 2) and mean percent ABCG2-positive cell populations (Table 1) was attained across the eight cell lines (*r *= 0.919, *P *= 0.001; Pearson product-moment correlation coefficient). There was, however, no correlation between relative quantity of mRNA for Bmi-1 and mean percent Bmi-1-positive cell populations (*r *= −0.311, *P *= 0.453; Pearson). The mild dysplasia-derived cell line (DOK) and an OSCC-derived cell line (PE/CA PJ15) were exceptional in having very high ABCG2 gene expression relative to control (OKF6-TERT2); another OSCC-derived cell line (SCC04) gave the lowest relative expression of ABCG2 (Table 2). Overall, there were no discernible trends between ABCG2 expression and source tissue disease severity (Fig. 2). For Bmi-1 gene expression, only the OSCC-derived SCC04 cell line exhibited lower Bmi-1 expression than control (OKF6-TERT2), all other cell lines exhibited two or three times higher Bmi-1 expression relative to control (Table 2), without displaying discernible trends between Bmi-1 expression and source tissue disease severity (Fig. 2).

**Table 2:** Mean (SD) relative quantity of mRNA (using OKF6-TERT2 as comparator) for ABCG2 and Bmi-1 expression in normal, dysplasia and SCC-derived oral cell lines by real-time PCR.

	Relative quantity mRNA (arbitrary units)	
	ABCG2	Bmi1
OKF6-TERT2	0.77 (0.55)	1.09 (0.45)
DOK	154.80 (15.76)	3.60 (0.49)
POE-9n	15.99 (2.44)	3.14 (0.31)
PE/CA PJ15	152.45 (20.01)	2.11 (0.19)
SCC04	0.41 (0.22)	0.38 (0.09)
SCC25	16.45 (2.23)	2.48 (2.07)
SCC09	29.77 (15.45)	3.41 (1.22)
SCC15	8.89 (2.29)	1.29 (0.21)

### ABCG2 and Bmi-1 antigen stain intensity in normal, dysplastic, and OSCC biopsies

**Figure 3:**
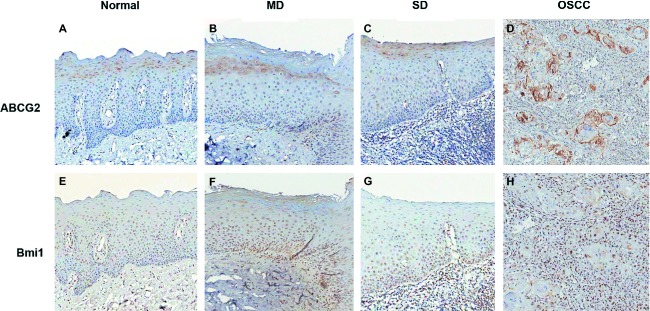
Representative immunostaining of normal, mild dysplasia, moderate-severe dysplasia and OSCC for Bmi-1, ABCG2 (original magnification ×100).

**Figure 4:**
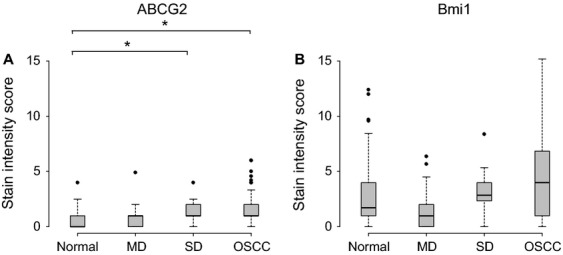
Box and Whisker plots of ABCG2 (A) and Bmi1 (B) immunoscore grouped by histopathology in normal oral mucosa, mild dysplasia (MD), severe dysplasia (SD), and OSCC cases showing median (bold line), 25th to 75th percentiles (box) and outliers (circles). *P < 0.016 (Mann–Whitney *U* test).

Representative immunostaining for ABCG2 and Bmi-1 are shown in Figure 3, and nonparametric descriptive statistics for ABCG2 and Bmi-1 immunoreactivity index scores are shown in Figure 4.

In all normal, dysplastic, and cancerous tissues, ABCG2 exhibited cytoplasmic localization with some membranous staining (Figs 3A–D). It was present in the stratum basale, stratum spinosum, and stratum granulosum with the vast majority of positive cells being in the supra-basal layers. Immunoreactivity for ABCG2 in normal tissue was weak giving a median index score of 0 (IQR = 0–1). Relative to normal tissue, ABCG2 immunoreactivity index scores were significantly elevated for moderate-severely dysplastic tissue (*P *= 0.004; Mann–Whitney) and OSCC tissue (*P *< 0.001; Mann–Whitney) only. Overall, ABCG2 antigen stain intensity increased with disease severity, although this trend was only modestly evident from the immunoreactivity index scores: mild dysplasia (median score = 1; IQR = 0–1) < moderate-severe dysplasia (median score = 1; IQR = 1–2) <OSCC tissue (median score = 1.1; IQR = 1–2; Fig. 4).

Bmi-1 was localized to the nucleus in all tissue grades (Figs 3E–H), and was expressed in the stratum spinosum and stratum basale only. Irrespective of tissue grade, immunoreactivity index scores were generally higher for Bmi-1 than those for ABCG2 (Fig. 4). None of the diseased tissue groups could be discriminated from normal tissue using Bmi-1 alone (*P *> 0.016; Mann–Whitney). Immunoreactivity for Bmi-1 was least intense for normal (median score = 1.7; IQR = 1–4) and mild dysplasia tissues (median score = 1; IQR = 0–2), higher for moderate-severe dysplasia (median score = 2.86; IQR = 2.33–4), and highest for OSCC tissues (median score = 4; IQR = 1–6.86), however, the intragroup range of immunoreactivity index scores mask any trend between Bmi-1 staining and disease severity. Across all tissue grades, correlation between ABCG2 and Bmi-1 immunoscores achieved statistical significance although the trend was numerically negligible (*r *= 0.144, *P *= 0.048; Pearson product-moment correlation coefficient).

## Discussion

In this study, we analyzed immunoreactive protein and relative gene expression of ABCG2 and Bmi-1 in a panel of eight cell lines derived from oral tissues with a spectrum of diseases ranging from normal through mild and moderate/severe dysplasia to oral squamous cell carcinoma (OSCC). In addition, we analyzed immunoreactive protein expression of ABCG2 and Bmi-1 gene products in an archive of 189 formalin-fixed paraffin-embedded (FFPE) patient oral tissue samples bearing the same range of disease severity as the cell lines. Our aim was to ascertain whether in vitro analysis with established cell lines provides a valid model for further investigation of the mechanistic involvement of ABCG2 and Bmi-1 in neoplastic transformation of oral dysplasia to OSCC, and to evaluate whether immunohistochemical analysis of ABCG2 and Bmi-1 has diagnostic potential for discriminating oral dysplasia from normal tissue or OSCC. Overall, the results indicate that cell populations that are positive for the putative cancer stem cell markers ABCG2 and Bmi-1 can occur with increased density in malignant or oral potentially malignant lesions, and cell lines derived from them. This indicates that carefully selected and maintained oral cell lines provide a valid model for investigating ABCG2 and Bmi-1 involvement in neoplastic transformation of oral dysplasia to OSCC, but that intragroup variability within IHC, flow cytometry, and RT-PCR results could compromise the diagnostic potential of the techniques for discriminating oral dysplasia from normal tissue or OSCC.

Flow cytometric analysis of oral lesion-derived cell lines gave a convincing correlation between source tissue disease severity and increased ABCG2 and Bmi-1 expression, yet even among five OSCC-derived cell lines there was considerable variability in the expression of ABCG2 and Bmi-1. Semi-quantitative real-time PCR results correlated well with mean percent positive cell population flow cytometry data for ABCG2 (*r *= 0.919, *P *= 0.001; Pearson) but not with Bmi-1 (*r *= −0.311, *P *= 0.453; Pearson) for the eight oral cell lines. Immunohistochemical study of FFPE patient biopsies demonstrated a limited trend between the diagnostic grading of oral lesion severity to ABCG2 and Bmi-1 immunostain intensity scores, the power of which was compromised by considerable intragroup variability. Association of ABCG2 and Bmi-1 with transformation of oral potentially malignant lesions to cancer has been demonstrated in a recent long-term follow-up study of 135 patients with oral leukoplakia (mean (SD) duration follow-up: 5.6 (3.7) years) 2. This earlier study reported increased risk of malignant transformation for lesions that were either ABCG2 positive (3.24-fold increase) or Bmi-1 positive (4.03-fold increase), and a statistically significant, although numerically negligible (*r *= 0.195), correlation between ABCG2 and Bmi-1 immunoscore (*P *= 0.24; Pearson correlation coefficient, 0.195) 2. Overall, the pattern of staining reported previously shows the same trend as reported here, with increased ABCG2 and Bmi-1 immunoscore coinciding with increased disease severity; and a statistically significant, although numerically negligible, correlation between ABCG2 and Bmi-1 expression across the entire cohort (*r *= 0.144, *P *= 0.048; Pearson product-moment correlation coefficient). We are unable to comment on the ability of ABCG2 and Bmi-1 to predict transformation of oral dysplasia tissues to OSCC because few of the patients were biopsied more than once, and we are unable to comment upon survival rates for the OSCC patient groups as these data lie beyond our ethical constraints.

Irrespective of the tissue diagnosis, immunoreactivity index scores were generally higher for Bmi-1 than ABCG2. The opposite trend was predominant for cell lines analyzed by flow cytometry, with five lines (OKF6-TERT2, POE9n, DOK, PE/CA PJ15 and SCC25) exhibiting larger proportions of ABCG2 positive than Bmi-1 positive populations, two lines (SCC09 and SCC15) exhibiting equivalent ABCG2 and Bmi-1 populations, and just one line (SCC04) exhibiting predominance of Bmi-1 over ABCG2. Difference between ex vivo and in vitro predominance of one antigen over another may reflect either an inherent feature of the cell lines or an influence of the cell culture conditions. Routine submerged monolayer culture tends to suppress oral epithelial cell differentiation, has the propensity to select for proliferative subpopulations and may preferentially select for cells with repressed differentiation pathways. Alternatively, organotypic culture of oral cell lines permits the establishment of tissue hierarchy and generates patterns of putative stem cell marker expression that are more representative of ex vivo tissues 23. In this study, cells for flow cytometry were harvested from subconfluent cultures in post logarithmic growth phase at which point the influence of contact inhibition on cellular growth and differentiation are minimal. Future related work should include study of organotypic cultures by IHC and rtPCR, noting that organotypic cultures are not readily compatible with flow cytometry.

This study was successful in using a diverse panel of oral cell lines to demonstrate a trend between source tissue disease severity and expression of the putative stem cell markers: ABCG2 and Bmi-1. Presence and absence of mRNA to protein correlation for ABCG2 and Bmi-1, respectively, indicated that rtPCR could be used in place of flow cytometry in future oral cell line-based studies of ABCG2 but not Bmi-1 expression. Confirmation of the trend between putative stem cell marker expression and oral lesion severity was achieved with a cohort of 189 FFPE patient biopsies. We observed discrepancy in ABCG2 versus Bmi-1 predominance between cell line and patient biopsy investigations which lead us to speculate whether organotypic 23 rather than monolayer culture may be preferable for future cell line-based investigations. Immunoreactivity index scoring of 189 patient biopsies revealed a trend between diagnostic grading of oral lesion severity and expression of the putative stem cell markers: ABCG2 and Bmi-1. These findings complement the previously reported potential for ABCG2 and Bmi-1 immunostaining to predict malignant transformation from oral leukoplakia 2, but our data indicate that intragroup variability within IHC, flow cytometry and RT-PCR results may compromise the diagnostic potential of these techniques for discriminating oral dysplasia from normal tissue or OSCC. Overall, the results provide evidence of increased density of ABCG2 and Bmi-1 positive populations in malignant and oral potentially malignant lesions and cell lines derived from them.

## Supporting Information

Figure S1 Positive control immunostaining for ABCG2 and Bmi1 in colon cancer tissue (original magnification × 200).

Figure S2 Representative scatter plots of ABCG2 and Bmi-1 expression in OKF6-TERT2, POE-9n, DOK, PE/ CA PJ15, SCC04, SCC25, SCC09, SCC15 cell lines maintained in submerged monolayer culture.
