# Supplementary figures and images for: Expression of ABCG2 and Bmi-1 in oral potentially malignant lesions and oral squamous cell carcinoma
Source: Cancer Med. 2014 Jan 11;3(2):273–83. doi: 10.1002/cam4.182 (PMC3987077; doi:10.1002/cam4.182)

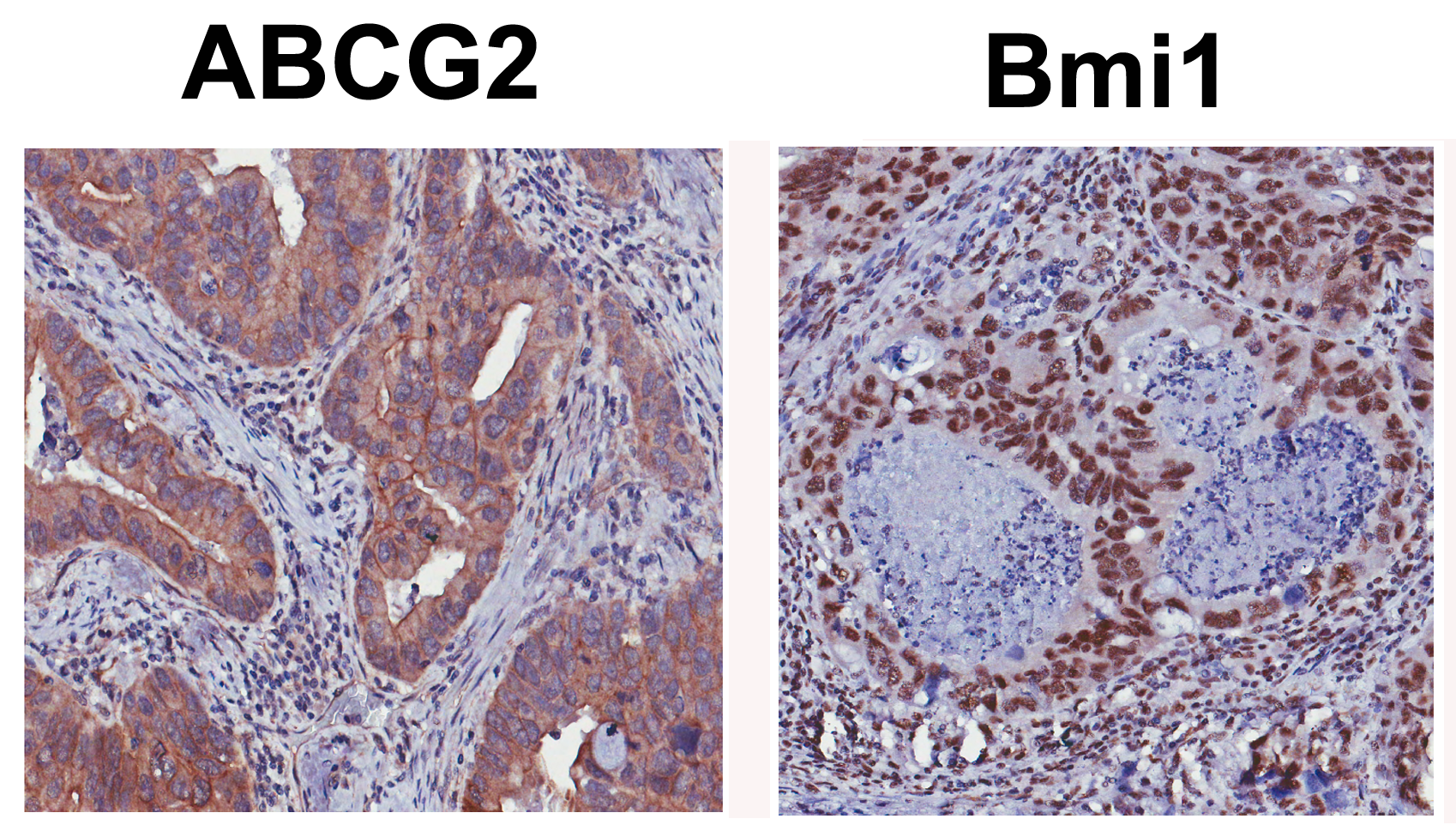

Supplement: Figure S1 — Positive control immunostaining for ABCG2 and Bmi1 in colon cancer tissue (original magnification × 200). [file cam40003-0273-sd1.tif]
