# Supplementary material for: Expression of ABCG2 and Bmi-1 in oral potentially malignant lesions and oral squamous cell carcinoma
Source: Cancer Med. 2014 Jan 11;3(2):273–83. doi: 10.1002/cam4.182 (PMC3987077; doi:10.1002/cam4.182)
Supplement: Figure S2 — Representative scatter plots of ABCG2 and Bmi-1 expression in OKF6-TERT2, POE-9n, DOK, PE/ CA PJ15, SCC04, SCC25, SCC09, SCC15 cell lines maintained in submerged monolayer culture. [file cam40003-0273-sd2.docx]

|  |  |
| --- | --- |
|  |  |
|  |  |

|  |  |
| --- | --- |
|  |  |
|  |  |

**Supplementary Figure 2**

Representative scatter plots of ABCG2 and Bmi-1 expression in OKF6-TERT2, POE-9n, DOK, PE/CA PJ15, SCC04, SCC25, SCC09, SCC15 cell lines maintained in submerged monolayer culture. Gate colours: Purple, ABCG2+/ Bmi-1 +; Blue, ABCG2- / Bmi-1+; Black, ABCG2+ / Bmi-1 -; Green, ABCG2- / Bmi-1-. Relevant isotype control histograms are provided in figure 3 of the manuscript.
